# Mindfulness and Balanced Time Perspective: Predictive Model of Psychological Well-Being and Gender Differences in College Students

**DOI:** 10.3390/ejihpe12030022

**Published:** 2022-03-10

**Authors:** Andrea Fuentes, Cristián Oyanadel, Philip Zimbardo, Melissa González-Loyola, Lening A. Olivera-Figueroa, Wenceslao Peñate

**Affiliations:** 1 Department of Psychology, Universidad de Concepcion, Concepcion 4030000, Chile; anfuentesp@udec.cl (A.F.); meligonzalez@udec.cl (M.G.-L.); 2 Department of Psychology, Stanford University, Stanford, CA 94305, USA; zim@stanford.edu; 3 Department of Psychiatry, School of Medicine, Yale University, New Haven, CT 06511, USA; lening.olivera@aya.yale.edu; 4 Department of Clinical Psychology, Psychobiology and Methodology, Universidad de La Laguna, 38200 La Laguna, Spain; wpenate@ull.edu.es

**Keywords:** mindfulness, balanced time perspective, psychological well-being

## Abstract

Background: The aims of the study were to establish an adjustment model to analyze the relationship among mindfulness, balanced time perspective (BTP) and psychological well-being (PWB) in college students and to explore gender differences among the variables. Method: The sample consisted of 380 college students, 220 women and 160 men, uniformly distributed according to the university’s faculties. Results: The results indicate that the synergy between mindfulness and BTP predicts the variance of PWB by 55%. Regarding gender differences, it was found that women have a greater tendency towards Past Positive than men and men a higher tendency towards Present Hedonistic than women. In addition, in the group of women, a stronger relationship was found among the variables and, consequently, a greater predictive value for PWB (58%), displaying an enhanced disposition to high PWB compared to men. Conclusions: Together, mindfulness and BTP promote optimal psychological functioning and alleviate or reduce discomfort. Thus, their promotion and training in universities is especially important given the high prevalence of anxiety and depressive symptoms in college students.

## 1. Introduction

The university population, being in a period of change and transition and facing high demands, represents one of the groups at the greatest risk for the development of mental health disorders and deterioration or abandonment of their academic activities [1].

A high prevalence of depressive (30%) and anxious symptoms (20–23%) was reported in the Chilean university population, which could be associated with academic stress, mental exhaustion perception and inability to respond to academic demands [2,3]. This prevalence was also observed in other countries. In the U.S., 26.6% of students reported depression and 8.2% suicidal ideation [4].

Given this, it is necessary to implement preventive interventions focused on promoting optimal psychological functioning and not just the absence of affliction. Mindfulness and balanced time perspective, then, emerge as cultivable, psychological resources that promote well-being [5,6] and can be extremely useful for college students. Research on the comparative efficacy of preventive interventions with a time perspective is scarce [7], but the results are promising for improving mental health, and it is suggested that restoring the temporal balance may be an indicator of health in the clinical context [8].

### 1.1. Mindfulness

Mindfulness refers to a non-judgmental state of attention and awareness of the present moment [9]. These capacities of attention and awareness are present, to a greater or lesser extent, in all human beings and can be cultivated and perfected by the practice of meditation [10,11]. Meditation is the process of observing body and mind, allowing synchronization with the present experiences that unfold moment by moment, accepting them as they are with a compassionate attitude, without judging and without reacting [12].

In recent times, mindfulness training has gained popularity thanks to its many benefits for health and well-being. In clinical contexts, mindfulness-based interventions (MBIs) relieve physical discomfort and pain [13]; improve psychological symptoms associated with depression and anxiety; reduce emotional reactivity and promote self-regulation; and show the same effectiveness on anxiety as cognitive behavioral therapies [14,15].

Mindfulness also promotes subjective and psychological well-being (PWB) [5], subjective happiness, positive affect [16], life satisfaction [17] and psychological flexibility [18], among other positive benefits for mental health.

Regarding gender differences in mindfulness, the findings are mixed and scarce. It was found that gender influences the level of mindfulness, with women tending to have lower levels of dispositional mindfulness than men [19], though no difference was found in other studies [20]. However, MBIs seem to be more beneficial for women. In an intervention for university students, women exhibited lower levels of stress and greater well-being than men post intervention [21]. Additionally, in a review of MBIs for substance abuse, a greater efficacy was found for women [22].

### 1.2. Balanced Time Perspective (BTP)

Time perspective refers to a process mediated by cultural, social and individual factors through which categories or time frames are assigned to personal and social events and experiences, which guide decision making and individual actions [23]. In the western world, five time orientations were identified: Past Positive, Past Negative, Present Hedonistic, Present Fatalistic and Future [24].

Balanced time perspective (BTP) refers to the process that allows individuals to flexibly switch between different time frames depending on the situation and personal resources. This balanced perspective includes positive attitudes towards the past, enjoyment of present experiences and the achievement of future goals [23] and can be promoted through group interventions [25] and psychotherapy [26].

BTP is associated with higher levels of subjective well-being, self-regulation [6], life satisfaction [27], higher rates of physical and mental health [28,29], resilience to psychological problems (anxiety, stress and depression) [30] and amplified cortisol reactivity to psychosocial stressors [31], among other benefits.

Findings on gender differences in time perspective are also mixed. Zimbardo et al. (1997) [32] reported that college men are more present-oriented than women and more hedonistically oriented [32]. As for females, higher scores in Positive Past and Future [23,33] were reported and higher scores in Present Fatalistic [34]. Likewise, lower scores were reported in Negative Future in women compared to men [35], whereas, in other studies, men were found to have a greater future orientation than women [36].

### 1.3. Mindfulness, Balanced Time Perspective and Psychological Well-Being

From a psychology of time point of view, mindfulness is considered as a holistic time perspective, associated with an awareness and attention towards the present in conjunction with an attitude oriented towards future goals and objectives, in contrast to the Present Hedonistic, which focuses on the search for immediate pleasure without accounting for future consequences [37].

Mindfulness and BTP are highly related time perspectives that promote positive mental health [38]. Thus, people with a balanced profile present higher levels of mindfulness [37,39]. Additionally, mindfulness is negatively related to both Past Negative and Present Fatalistic and positively related with both Past Positive and Future [37,40]. In addition, BTP was found to be a partial positive mediator between mindfulness and life satisfaction [17].

Many studies reported that mindfulness skills promote higher levels of PWB [16,41,42]. PWB was understood as a more stable, optimal psychological functioning that arises from the development of human potential, distinguishing itself from subjective well-being which is defined in terms of happiness and life satisfaction, tending to be a more ephemeral state [43,44,45].

Mindfulness tends to be more strongly associated with PWB than with subjective well-being [5], and it was found that the relationship between the two is mediated by clarity in self-concept [46], fewer attempts to suppress certain thoughts and emotional self-regulation in female college students [47].

The relationship between BTP and PWB has scarcely been studied. However, positive relationships were found between both constructs [48]. For instance, in young adults, higher levels of PWB were found to be positively associated with Past Positive and Future and negatively associated with Past Negative and Present Fatalistic [49]. 

As such, the relationship among the three constructs requires further investigation, especially that between BTP and PWB. Thus, the present study aims to: (1) establish an adjustment model to analyze the relationship among mindfulness, BTP and PWB in the university population and (2) make an exploratory analysis regarding gender differences in mindfulness, BTP and PWB.

Because a close positive relationship pattern was expected among mindfulness, BTP and PWB, we hypothesized the existence of a predictive model where, individually, mindfulness and BTP and their synergy provide a strong contribution in the prediction of PWB. In addition, because gender differences were expected, that model was differentially expressed for female and male. 

Results obtained through this research could be useful as a theoretical contribution that supports and motivates the promotion of mindfulness training programs and a balanced time perspective in universities which, in turn, could stimulate optimal mental health, well-being and the generation of protective factors in college students.

## 2. Materials and Methods

### 2.1. Participants

The sample consisted of a total of 380 students (220 women, 160 men) from the Universidad de Concepción (Chile), ages ranging from 18 to 25 years old. Regarding their economic status, 42.4% were in the lower-income, 37.9% in the middle-income and 19.7% in the high-income population. The students participated voluntarily, and their selection was non-probabilistic and by convenience. The initial sample was 407 participants, of which 27 were randomly selected to obtain a uniform, stratified sample of 20 participants for each of the 19 faculties of the Universidad de Concepción. The data set aside were used later to validate the regression model.

### 2.2. Instruments

Five Facets of Mindfulness Questionnaire (FFMQ; Baer et al., 2006), validated in Chile by Schmidt and Vinet (2015) [50]. The questionnaire consists of 39 items scored on a Likert scale in a range from 1 (never or very rarely true) to 5 (very often or always true). The FFMQ measures five facets of mindfulness: (1) Observe: capacity for paying attention or perceiving internal and external stimuli; (2) Describe: noticing or labeling these stimuli with words; (3) Act with Awareness: acting or attending to the present moment instead of automatically or with an absent mind; (4) Non-Judging Internal Experience: refraining from evaluating one’s sensations, cognitions and emotions; and (5) Non-Reactivity to Internal Experience: allowing thoughts and feelings to come and go without attention being drawn into them [51].

Regarding reliability, in this study, the total internal consistency was adequate (α = 0.86), as was the case across the facets: Observe (α = 0.76), Describe (α = 0.87), Act with Awareness (α = 0.87), Non-Judging (α = 0.86) and Non-Reactivity (α = 0.69). 

Scales of Psychological Well-Being (SPWB) [45], adapted to Spanish by Díaz et al. (2006) [52]. Its Spanish version consists of 29 items scored in a range from 1 (totally disagree) to 5 (totally agree). SPWB measures six dimensions of psychological well-being: (1) Self-Acceptance: which refers to having positive attitudes towards oneself, accepting oneself now, as well as the past. It is one of the fundamental criteria of well-being and a characteristic of self-actualization, optimal functioning and maturation; (2) Positive Relations with Others: ability to interact and have positive and stable links with others; (3) Autonomy: ability to settle on one’s own convictions, maintaining one’s independence and personal authority in various social contexts; (4) Environmental Mastery: ability to choose or create optimal environments to satisfy one’s own wants and needs, feeling in control and able to influence what happens to one; (5) Purpose in Life: establishing goals that give meaning to one’s life; and (6) Personal Growth: encompassing the need of the human being to develop personal capacities, maintaining determination to continue growing as a person and maximizing potential.

Ryff et al. (1995; 2008) [53,54] reported that well-being varies with age. Autonomy and Environmental Mastery tend to increase, while Purpose in Life and Personal Growth tend to decrease. When it comes to gender differences, the results were mixed. Ryff et al. (1995) [53] reported that only Positive Relations with Others had gender differences, with women scoring higher than men. Other studies reported that women had higher scores than men in Environmental Mastery [55], Personal Growth, Positive Relations with Others and Self-Acceptance [56].

Regarding reliability, in this study, a high internal consistency was obtained with a Cronbach’s alpha of 0.91, as in the 6 dimensions: Self-Acceptance (α = 0.87), Autonomy (α = 0.68), Positive Relations with Others (α = 0.76), Environmental Mastery (α = 0.68), Purpose in Life (α = 0.81) and Personal Growth (α = 0.69).

Zimbardo Time Perspective Inventory (ZTPI; Zimbardo and Boyd, 1999 [23]), adapted and validated in Chile by Oyanadel, Buela-Casal and Pérez-Fortis (2014) [57]. This study used the reduced version developed by Jofré et al. (2021) [58], which consists of 15 items with a 5-point Likert scale. Regarding reliability, this version shows an adequate internal consistency on all the scales: Past Positive (α = 0.51), Past Negative (α = 0.71), Present Hedonistic (α = 0.69), Present Fatalistic (α = 0.64) and Future (α = 0.69). 

The ZTPI measures five time perspectives: (1) Past Positive: reflects a positive, pleasurable, often nostalgic view of the past, focusing on maintaining positive relationships with family and friends; (2) Past Negative: negative view of the past, focusing mainly in aversive or noxious situations. It is reflected in conservative and cautious conduct; (3) Present Hedonistic: orientation toward the hedonistic pleasures without concern about consequences; (4) Present Fatalistic: reflects hopelessness about the present and belief in external forces that controls one’s life and (5) Future: orientation toward the achievement of future goals and outcomes of present actions and decisions [59].

### 2.3. Temporal Profiles Definition

Deviation from the balanced time perspective (DBTP; [60]) is an equilibrium index in time perspective, measured through the ZTPI. Scores are calculated from the square root of the sum of the squared difference between the optimal and individual scores of Past Negative (ePN), Past Positive (ePP), Present Fatalistic (ePF), Present Hedonistic (ePH) and Future (eF). The calculation being the following:

$$\mathrm{DBTP}=\sqrt{{\left(1.95-\mathrm{ePN}\right)}^{2}+{\left(4.6-\mathrm{ePP}\right)}^{2}+{\left(1.5-\mathrm{ePF}\right)}^{2}+{\left(3.9-\mathrm{ePH}\right)}^{2}+{\left(4-\mathrm{eF}\right)}^{2}}$$

### 2.4. Procedure

#### Data Collection and Ethical Considerations

The instrument’s application was carried out by an online questionnaire, as it allowed easier and quicker access for university students. The participants were invited via social media, where the link was published. To ensure that the participants where from the university, their institute’s email was requested.

In parallel, face-to-face instruments were applied. For this, the participants were recruited and tested in small groups at their faculties in between classes. For ethical considerations, each of the participants was asked for their informed consent prior to the application of the instruments, both online and in person.

### 2.5. Data Analysis

Statistical analysis was performed using IBM SPSS Statistics for Windows, version 25.0 (IBM Corp., Armonk, NY, USA). To address the relationships among mindfulness, BTP and PWB, a Pearson correlational analysis was performed among FFMQ, ZTPI and SPWB and the deviation from the balanced time perspective (DBTP). Subsequently, in order to know the predictive value of mindfulness and BTP on PWB, a stepwise regression was carried out. The model obtained was verified in real data previously extracted from the analyzed sample. Regarding the exploration of gender differences between the variables, Pearson’s correlations were used differently between genders, and a multivariate analysis of variance (MANOVA) was performed in order to compare the means in the study variables. Finally, the same predictive model of differentiated PWB was carried out in women and men, and it was verified in the data extracted, as well as in the general model.

## 3. Results

The correlations among the FFMQ, ZTPI and DBTP are shown in Table 1. The results indicate that the mindfulness overall score measured through the FFMQ was positively related to Future and Past Positive and negatively to Past Negative and Present Fatalistic. No significant relationship was found with Present Hedonistic. The DBTP was negatively related to the overall FFMQ score and to most of its facets with the exception of Observe.

Table 2 shows the correlations among the SPWB, FFMQ and the temporal perspectives. As expected, the PWB and FFMQ overall score were positively related, as well as its dimensions and facets with the exception of the Autonomy dimension with the Observe facet. The PWB overall score was negatively related to DBTP, Present Fatalistic and Past Negative, and it was positively related to the Future and Past Positive.

**Figure 1:**
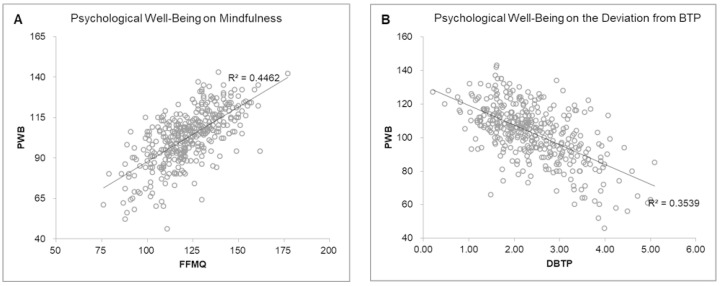
Scatterplots of psychological well-being determined by (**A**) mindfulness and (**B**) deviation from balanced temporal perspective.

Regarding the calculation of the predictive value of mindfulness (FFMQ) and BTP on PWB, a linear regression was performed with the mindfulness overall score and DBTP due to the high correlation shown between them and the overall score of PWB. It was obtained that mindfulness explained 45% of the variance of PWB, while DBTP explained 35% (Figure 1).

Since the explanatory variables presented a linear relationship, a stepwise regression methodology was used, obtaining the following adjustment model for the PWB variable:

$$\hat{\mathrm{PWB}}=57.994+0.506\;\left(\mathrm{FFMQ}\right)-7.151\;\left(\mathrm{DBTP}\right)$$

Thus, PWB increases with the increase of mindfulness and the decrease of the DBTP; that is, when tending towards a balanced time perspective. As expected, the synergy between FFMQ and DBTP had a greater predictive power than individually, determining the variance of PWB by 55% (R^2^ = 0.548, F (2,377) = 230.7, *p* < 0.001). 

**Figure 2:**
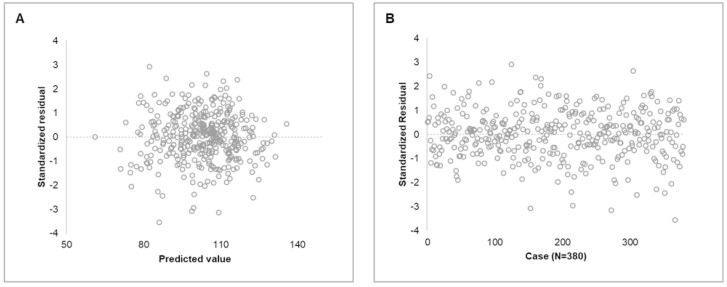
Scatter plots of the standardized residuals. (**A**) standardized residuals vs. the predicted value; (**B**) standardized residuals vs. the sample cases (*N* = 380).

The goodness of fit analysis of the model for the standardized residuals using the Kolmogorov–Smirnov test determined that the residuals presented a normal distribution. The scatter plots of the residuals are presented in Figure 2, showing that they did not present any pattern and maintained a mean 0 with a constant standard deviation.

Finally, the model was verified in data independent from the sample used for the development of it. It was obtained that 70% (19 of 27) of these fitted the model, locating the estimated PWB values in the same quintiles as the observed values.

### Gender Differences

To evaluate gender differences in mindfulness, temporal perspectives and PWB scores, a multivariate analysis of variance (MANOVA) was performed. Table 3 presents the gender differences for each variable. Using the Wilk’s lambda criteria, the test was statistically significant (F (17,362) = 2.612; *p* < 0.001). Women exhibited higher scores in Past Positive compared to men, whereas men showed a greater tendency towards the Present Hedonistic and higher scores in Non-Reactivity compared to women.

A Pearson correlation analysis between the variables in the group of women and men separately showed that women presented higher correlations between the scores of FFMQ, ZPTI and SPWB than the overall sample (Table 4). These results could be interpreted as indicative that, among females, mindfulness and BTP have a greater power over PWB.

Finally, a stepwise regression was carried out to explore differences in the predictive model of PWB between the group of women and men. The level of mindfulness in women explained 51% of the variance of PWB, whereas, in men, only 32%. By adding DBTP, in women, a predictive value of R^2^ = 0.578 (F (2,217) = 150.94, *p* < 0.001) was obtained, greater than the total sample, while, in men, a predictive value of R^2^ = 0.488 (F (2,157) = 76.77, *p* < 0.001) was obtained.

The adjustment model in the group of women had a higher predictive value for PWB, this being the following:

$$\hat{\mathrm{PWB}}=49.692+0.564\;\left(\mathrm{FFMQ}\right)\;-\;6.658\;\left(\mathrm{DBTP}\right)$$

Thus, like the model for the overall sample of university students, this model indicates that, in women, PWB increases when mindfulness increases and DBTP decreases.

The goodness of fit analysis of the model indicated that, by means of the Kolmogorov–Smirnov test, the standardized residuals had a normal distribution. Using the data set aside at the beginning of the study, it was obtained that 10 out of 18 (55.6%) participants in the group of women fitted the model, locating the estimated PWB values in the same quintiles as those observed. This model had a lower fit than the one of the overall sample, probably due the smaller data in which it was contrasted. It is important to emphasize that the data used for this purpose were independent and did not contribute to the development of the model. Thus, it is necessary for further research to study the stability of this model in a larger data-set.

## 4. Discussion

The aims of this study were to establish an adjustment model to analyze the relationship between mindfulness, BTP and PWB in a university population and to explore the possible gender differences regarding the study variables. As was expected, mindfulness was positively related to BTP, since the greater the tendency towards a balanced profile and the lower the tendency towards a NTP, the greater the level of mindfulness. These findings are in agreement with those reported by Drake et al. (2008) [39], Muro et al. (2017) [40], Seema and Sircova (2013) [37] and Stolarski et al. (2016) [17]. However, no relationship was found between BTP and the Observe facet of mindfulness.

Similarly, a positive relationship was found between mindfulness and the TP orientations Past Positive and Future, as well as a negative relationship between mindfulness and the TP orientations Past Negative and Present Fatalistic. These results observed in our study are in congruence with the results obtained by Seema and Sircova (2013) [37] and Muro et al. (2017) [40]. 

Regarding PWB, a positive relationship was found between BTP and PWB, showing that the greater the trend towards a balanced time profile, the greater the PWB. This finding is in line with the findings reported by García et al. (2016) [48] and Zambianchi (2019) [49], where PWB and its dimensions were found to be positively related to Past Positive and Future, as well as negatively related to Past Negative and Present Fatalistic. Moreover, the dimensions of Self-Acceptance, Mastery of the Environment and Purpose in Life also appeared to be positively and moderately related to Past Positive and Future, as well as negatively related to Past Negative and Present Fatalistic. 

Similarly, in our study, mindfulness and PWB appeared to have a strong positive relationship, which is in agreement with previous studies [5,16,41,42,46,47]. As such, those students who are capable of describing present, external and internal stimuli and acting on them consciously are more likely to accept themselves, set goals in life and perceive control over their environment.

Regarding the adjustment model among mindfulness, BTP and PWB, it was found that mindfulness and BTP together determined 55% of the variance in PWB. This model was initially validated on a separate data-set that did not contribute to the development of the study and demonstrated a 70% fit, corroborating that both constructs promote optimal psychological functioning and not only alleviate or reduce the discomfort, but also generate a more stable, positive effect over time.

It is important, then, for the processes of personal development and self-actualization, to have a state of attention and full consciousness that allows accepting and enjoying the present as it is presented moment by moment, to have a positive and accepting attitude towards past experiences and to establish future goals, generating actions in the present to achieve them. 

Regarding gender, significant differences were found in time perspective, with men presenting higher scores in Present Hedonistic, which is consonant with the findings of D’Alessio et al. (2003) [34]. Likewise, women presented higher scores in the Past Positive in the present study, which also stands in agreement with previous research [23,34]. Thus, men tend to exhibit more hedonistic behaviors in pursuit of immediate gratification, whereas women exhibit a more positive mentality and attitude towards the past. 

A stronger relationship was found between mindfulness, balanced time perspective and PWB in the group of women compared to men. The women’s predictive model of PWB exhibited a better fit, with mindfulness and BTP predicting PWB to a greater extent (58%). This may imply that mindfulness training interventions and promotion of a BTP are more effective and beneficial for the PWB of college women, and further research should be carried out in this regard.

In this case, the model had a 55.5% fit, lower in comparison to the population model, possibly due to the low number of participants in the data in which the model was contrasted. Thus, the fit of the model, as is the population’s, is highly susceptible to minimum variation.

### 4.1. Practical Implications

These results provide a theoretical basis for the generation of mental health promotion and prevention programs tailored to university population based on mindfulness training and the promotion of balance between temporal perspectives. Rönnlund et al. (2019) [61] reported that, by increasing levels of mindfulness through training, a more balanced perspective can be promoted. Previous research showed that group MBIs in college students are highly beneficial in reducing depression and anxiety symptoms, as well as in increasing life satisfaction [62], helping emotion regulation, increasing levels of mindfulness [63], enhancing the adjustment to college transition and reducing stress [64].

It is expected that mindfulness and time-perspective-based programs will generate relief for stress and anxiety symptoms and also help acquiring resources and protective factors that promote optimal mental health, which will allow students to cope more adaptively with stressful situations. This becomes especially relevant when taking into account the current pandemic context due to COVID-19, which has greatly affected people’s mental health, generating an increase in anxiety symptoms, stress and depression [65]. As such, the proposed programs could be implemented through online platforms, which have been demonstrated to be equally effective [66].

### 4.2. Future Directions

It is necessary for future research to address the stability of the predictive models across both women and the overall population, as well as through a larger data-set. 

In addition, it is suggested that further studies address the effects of mindfulness and BTP training programs on PWB, specifically its difference between women and men. Additionally, it is suggested that studies are carried out on factors that mediate the relationship between BTP, mindfulness and PWB.

### 4.3. Limitations

Regarding the limitations of the study, its main one is its cross-sectional nature, wherein a causal relationship between PWB and the synergy of mindfulness with BTP could not be affirmed. Second, there were different conditions and formats for applying the questionnaires, online and in person, which could have generated differences in the results. Third, the online application of the battery of instruments did not allow control over the application variables, unlike the face-to-face application.

## 5. Conclusions

This study demonstrates the existence of a positive relationship between mindfulness, BTP and PWB. Moreover, it establishes that mindfulness and BTP have a predictive power over PWB in a university population. Regarding gender differences, the relationship between the variables is expressed differentially between men and women. In college women the adjustment model exhibits a greater predictive value for PWB. Furthermore, we can conclude that college women experience an enhanced disposition to PWB compared to college men. It appears that, together, mindfulness and BTP promote optimal psychological functioning beyond alleviating or reducing discomfort. Thus, their promotion and training in universities is especially important given the high prevalence of anxiety and depressive symptoms in college students.

## Figures and Tables

**Table 1:** Correlations among FFMQ, its facets and ZTPI’s temporal perspectives.

	Five Facets of Mindfulness Questionnaire (FFMQ)					
ZPTI	Mindfulness Overall Score	NJ	NR	OB	D	AA
F	0.29 **	0.09	0.11 *	0.05	0.25 **	0.32 **
PP	0.37 **	0.14 **	0.14 **	0.17 **	0.26 **	0.34 **
PH	0.00	−0.07	0.10 *	0.23 **	0.02	−0.23 **
PN	−0.46 **	−0.47 **	−0.22 **	0.02	−0.25 **	−0.35 **
PF	−0.31 **	−0.21 **	−0.16 **	0.03	−0.23 **	−0.29 **
DBTP	−0.46 **	−0.31 **	−0.26 **	−0.10	−0.32 **	−0.35 **

Note. ** *p* < 0.01; * *p* < 0.05. NJ, Non-Judging; NR, Non-Reactivity; OB, Observe; D, Describe; AA, Act with Awareness; F, Future; PP, Past Positive; PH, Present Hedonistic; PN, Past Negative; PF, Present Fatalistic.

**Table 2:** Correlations between SPWB, FFMQ and ZTPI’s temporal perspectives.

	Scales of Psychological Well-Being (SPWB)						
	PWB Overall Score	SA	PR	AU	EM	PL	PG
FFMQ							
Mindfulness overall score	0.67 **	0.60 **	0.39 **	0.46 **	0.59 **	0.53 **	0.48 **
NJ	0.38 **	0.39 **	0.26 **	0.32 **	0.36 **	0.22 **	0.18 **
NR	0.34 **	0.31 **	0.18 **	0.21 **	0.33 **	0.28 **	0.25 **
OB	0.19 **	0.14 **	0.13 *	0.08	0.10 *	0.14 **	0.31 **
D	0.54 **	0.46 **	0.34 **	0.40 **	0.44 **	0.45 **	0.36 **
AA	0.47 **	0.41 **	0.22 **	0.29 **	0.47 **	0.44 **	0.30 **
ZTPI							
F	0.39 **	0.35 **	0.17 **	0.17 **	0.39 **	0.45 **	0.28 **
PP	0.45 **	0.47 **	0.27 **	0.11 *	0.44 **	0.47 **	0.30 **
PH	0.09	0.11 *	0.10 *	0.03	0.07	0.00	0.11 *
PN	−0.53 **	−0.50 **	−0.34 **	−0.34 **	−0.53 **	−0.37 **	−0.35 **
PF	−0.39 **	−0.34 **	−0.24 **	−0.13 *	−0.39 **	−0.39 **	−0.31 **
DBTP	−0.60 **	−0.60 **	−0.36 **	−0.24 **	−0.57 **	−0.52 **	−0.45 **

Note. ** *p* < 0.01; * *p* < 0.05. SA, Self-Acceptance; PR, Positive Relations with Others; AU, Autonomy; EM, Environmental Mastery; PL, Purpose in Life; PG, Personal Growth; NJ, Non-Judging; NR, Non-Reactivity; OB, Observe; D, Describe; AA, Act with Awareness; F, Future; PP, Past Positive; PH, Present Hedonistic; PN, Past Negative; PF, Present Fatalistic.

**Table 3:** Mean (M) and standard deviation (SD) for woman and men and gender differences for each measured variable.

	Women (*n* = 220)		Men (*n* = 160)		Gender Difference
	M	SD	M	SD	F (17, 362)
FFMQ					
Overall score	121.82	17.25	123.43	14.96	0.344
NJ	22.57	6.40	22.86	6.26	0.660
NR	21.58	4.12	22.77	4.11	0.006 **
OB	28.61	5.20	28.34	5.34	0.622
D	25.16	6.63	26.09	5.27	0.141
AA	23.90	6.14	23.36	5.86	0.395
ZTPI					
F	3.71	0.67	3.72	0.58	0.938
PP	3.85	0.77	3.62	0.75	0.004 **
PH	3.02	0.80	3.20	0.79	0.028 *
PN	3.11	0.84	3.14	0.91	0.719
PF	2.32	0.68	2.34	0.72	0.877
DBTP	2.36	0.85	2.41	0.83	0.546
PWB					
Overall score	102.65	17.96	103.42	14.38	0.653
SA	14.10	3.83	14.48	3.34	0.315
PR	17.96	4.30	17.68	3.44	0.484
AU	20.15	4.41	20.83	3.48	0.103
EM	16.97	3.49	17.43	3.28	0.198
PL	17.59	4.05	17.24	3.60	0.394
PG	15.89	2.95	15.77	2.79	0.695

Note. ** *p* < 0.01; * *p* < 0.05. NJ, Non-Judging; NR, Non-Reactivity; OB, Observe; D, Describe; AA, Act with Awareness; F, Future; PP, Past Positive; PH, Present Hedonistic; PN, Past Negative; PF, Present Fatalistic; SA, Self-Acceptance; AU, Autonomy; EM, Environmental Mastery; PL, Purpose in Life; PG, Personal Growth.

**Table 4:** Correlations between FFMQ, ZTPI’s temporal perspectives and SPWB in the group of university women.

	Mindfulness Overall Score	NJ	PWB Overall Score	SA	AU	EM	PL	PG
FFMQ								
Overall score			0.72 **	0.69 **		0.67 **	0.60 **	0.49 **
NJ				0.47 **				
D			0.64 **	0.57 **	0.50 **	0.57 **	0.56 **	
AA			0.50 **	0.48 **		0.53 **	0.48 **	
ZTPI								
F							0.47 **	
PP			0.51 **	0.55 **		0.53 **	0.51 **	
PN	−0.52 **	−0.49 **	−0.54 **	−0.53 **		−0.53 **		
PF							−0.46 **	
DBTP	−0.56 **		−0.62 **	−0.62 **		−0.62 **	−0.55 **	−0.46 **

Note. ** *p* < 0.01. SA, Self-Acceptance; AU, Autonomy; EM, Environmental Mastery; PL, Purpose in Life; PG, Personal Growth; NJ, Non-Judging; D, Describe; AA, Act with Awareness; F, Future; PP, Past Positive; PP, Past Negative; PF, Present Fatalistic.

## Data Availability

The data-sets generated and analyzed during the current study are available in Figshare repository, https://doi.org/10.6084/m9.figshare.14342315.v1, accessed on 20 January 2022.
